# Occupational patterns of opioid-related harms comparing a cohort of formerly injured workers to the general population in Ontario, Canada

**DOI:** 10.17269/s41997-024-00882-w

**Published:** 2024-04-24

**Authors:** Nancy Carnide, Gregory Feng, Chaojie Song, Paul A. Demers, Jill S. MacLeod, Jeavana Sritharan

**Affiliations:** 1 Institute for Work & Health, Toronto, Ontario Canada; 2 Dalla Lana School of Public Health, University of Toronto, Toronto, Ontario Canada; 3 Occupational Cancer Research Centre, Ontario Health, Toronto, Ontario Canada

**Keywords:** Drug overdose, Opioid-related disorders, Occupational injuries, Workplace, Workers’ compensation, Humans, Surdose d’opioïdes, désordres liés aux opioïdes, accidents du travail, lieu de travail, demandes d’indemnisation des accidentés du travail, humains

## Abstract

**Objectives:**

The role of work-related injuries as a risk factor for opioid-related harms has been hypothesized, but little data exist to support this relationship. The objective was to compare the incidence of opioid-related harms among a cohort of formerly injured workers to the general population in Ontario, Canada.

**Methods:**

Workers’ compensation claimants (1983–2019) were linked to emergency department (ED) and hospitalization records (2006–2020). Incident rates of opioid-related poisonings and mental and behavioural disorders were estimated among 1.7 million workers and in the general population. Standardized incidence ratios (SIRs) and 95% confidence intervals (CI) were calculated, adjusting for age, sex, year, and region.

**Results:**

Compared to the general population, opioid-related poisonings among this group of formerly injured workers were elevated in both ED (SIR = 2.41, 95% CI = 2.37–2.45) and hospitalization records (SIR = 1.54, 95% CI = 1.50–1.59). Opioid-related mental and behavioural disorders were also elevated compared to the general population (ED visits: SIR = 1.86, 95% CI = 1.83–1.89; hospitalizations: SIR = 1.42, 95% CI = 1.38–1.47). Most occupations and industries had higher risks of harm compared to the general population, particularly construction, materials handling, processing (mineral, metal, chemical), and machining and related occupations. Teaching occupations displayed decreased risks of harm.

**Conclusion:**

Findings support the hypothesis that work-related injuries have a role as a preventable risk factor for opioid-related harms. Strategies aimed at primary prevention of occupational injuries and secondary prevention of work disability and long-term opioid use are warranted.

**Supplementary Information:**

The online version contains supplementary material available at 10.17269/s41997-024-00882-w.

## Résumé

**Objectifs:**

Le rôle des accidents du travail en tant que facteur de risque pour les méfaits liés aux opioïdes a été supposé, mais il existe peu de données pour étayer cette relation. L’objectif était de comparer l’incidence des méfaits liés aux opioïdes dans une cohorte de travailleurs anciennement blessés à la population générale de l’Ontario, au Canada.

**Méthodes:**

Les dossiers de demandes d’indemnisation des travailleurs (1983‒2019) ont été reliés aux dossiers des services d’urgence et d’hospitalisation (2006‒2020). Les taux d’incidence des empoisonnements liés aux opioïdes et des troubles mentaux et comportementaux ont été estimés parmi 1,7 million de travailleurs et dans la population générale. Les ratios d’incidence standardisés (RIS) et les intervalles de confiance (IC) à 95 % ont été calculés en tenant compte de l’âge, du sexe, de l’année et de la région.

**Résultats:**

Comparativement à la population générale, les empoisonnements liés aux opioïdes chez ce groupe de travailleurs anciennement blessés étaient élevés dans les dossiers des services d’urgence (RIS = 2,41, IC à 95 % = 2,37-2,45) et d’hospitalisation (RIS = 1,54, IC à 95 % = 1,50-1,59). Les troubles mentaux et comportementaux liés aux opioïdes étaient également élevés par rapport à la population générale (visites aux urgences RIS = 1,86, IC à 95 % = 1,83-1,89; hospitalisations RIS = 1,42, IC à 95 % = 1,38-1,47). La plupart des professions et des secteurs d’activité présentaient des risques de méfaits plus élevés par rapport à la population générale, en particulier la construction, la manutention, le traitement (minéral, métallique, chimique) et l’usinage des matières premières et activités connexes. Les professions de l’enseignement présentaient des risques de méfaits moindres.

**Conclusion:**

Les résultats confirment l’hypothèse selon laquelle les accidents du travail constituent un facteur de risque évitable pour les méfaits liés aux opioïdes. Des stratégies visant la prévention primaire des accidents du travail et la prévention secondaire de l’incapacité de travail et de la consommation d’opioïdes à long terme sont justifiées.

## Introduction

An opioid crisis characterized by high rates of opioid-related harms continues to unfold across North America. Rates of opioid-related poisonings and use disorders have increasingly worsened, particularly since the onset of the COVID-19 pandemic (Ahmad et al., 2024; Federal, provincial, and territorial Special Advisory Committee on the Epidemic of Opioid Overdoses, 2023; Gomes et al., 2023; Hutchinson et al., 2023). Between January 2016 and June 2023 alone, 40,642 opioid toxicity deaths occurred in Canada and approximately 500,000 opioid-related deaths occurred in the United States (USA) over a similar time period (Federal, provincial, and territorial Special Advisory Committee on the Epidemic of Opioid Overdoses, 2023; Ahmad et al., 2024).

In both Canada and the USA, anywhere from 20% to 40% of opioid-related deaths with available employment status information have occurred among working adults (BC Coroners Service Death Review Panel, 2022; Gomes et al., 2022; Aram et al., 2020; Altekruse et al., 2020). The role of work-related injuries and pain as a determinant of opioid-related harms has been previously hypothesized (Shaw et al., 2020). Consistent with this hypothesis, individuals in traditionally blue-collar, labour-intensive occupations, such as construction and trades, transportation, manufacturing, maintenance and repair, and natural resource occupations, are known to be at greater risk for occupational injuries and musculoskeletal pain and have been found to have a high prevalence of opioid-related mortality (Aram et al., 2020; Chalasani et al., 2020; Hawkins et al., 2019; Harduar Morano et al., 2018; Scagos et al., 2019; BC Coroners Service Death Review Panel, 2022; Gomes et al., 2022; Billock et al., 2023). In one study, occupations classified as having high injury rates also exhibited higher rates of opioid-related deaths (Hawkins et al., 2021). Studies have also demonstrated opioid prescribing to be common after work-related injuries, including long-term prescriptions (Durand et al., 2019; Asfaw et al., 2022; Rosenman & Wang, 2022).

Studies conducted specifically among injured workers have found an association between duration of work disability and elevated rates of opioid-related deaths (Applebaum et al., 2019; Martin et al., 2020). One study of workers’ compensation claimants also found maximum opioid dose per claim to be associated with a greater risk of death, although cause of death data were not available (Freeman et al., 2022). Yet, few studies have empirically examined the role of work-related injuries in opioid-related morbidity and mortality. A descriptive study in Utah examining the characteristics of decedents of opioid-related deaths found over half (57%) had experienced at least one work-related injury in their lifetime (Cheng et al., 2013). More recently, a retrospective study in the USA using employer-sponsored health insurance data found injured workers to have 1.79 times the risk of opioid-related morbidity (diagnosed opioid abuse, dependence, adverse effects, or poisoning) compared to non-injured workers within the first 36 months of injury (Asfaw & Boden, 2020). Finally, a study conducted in West Virginia, comparing injured workers with low back pain workers’ compensation claims to the general population, found mortality from accidental poisoning (primarily due to opioids) was significantly elevated among the overall cohort of injured workers compared to the general population (standardized mortality ratio [SMR] = 1.62) (Martin et al., 2020). Little is known beyond these limited data and to our knowledge, differential risks by occupation have not been explored.

We aimed to further extend this research by comparing the incident rates of opioid-related harms, specifically poisonings and mental and behavioural disorders, among a cohort of formerly injured workers to those in the general population in Ontario, Canada, overall and by occupation and industry. A subgroup analysis by sex was also completed.

## Methods

### Study cohort and data sources

This study uses data from the Occupational Disease Surveillance System (ODSS). The ODSS was developed to monitor work-related diseases among workers in Ontario, Canada, through a set of linked administrative health databases. The ODSS was initially constructed using Ontario Workplace Safety and Insurance Board (WSIB) accepted lost-time workers’ compensation claims records from 1983 to 2019 for 2,368,218 workers. The WSIB is the provincial workers’ compensation insurer in Ontario, providing coverage to approximately 70‒75% of workers. Workers in the ODSS have been linked to records in the Ontario Health Insurance Plan’s Registered Persons Database (RPDB) (1990–2022), which includes demographic information, death date (where applicable), and residence, as well as a unique identifier, known as the Health Insurance Number (HIN) for all Ontarians registered for provincial health insurance. Linkage to the RPDB involved deterministic linkage methods, matching on exact criteria and specific personal identifiers (i.e., full name, date of birth, sex), and probabilistic linkage methods, with matching based on the likelihood that records correspond to the same worker (i.e., partial names or incomplete information). A total of 1,973,312 workers were successfully matched to an RPDB record with a corresponding HIN. Using the HIN, these workers were successfully linked using deterministic linkage to hospitalization records in the Discharge Abstract Database (DAD) and emergency department records in the National Ambulatory Care Reporting System (NACRS) (2006‒2020). Those without a HIN were excluded from the analysis (*n* = 394,906), as the HIN was required to match workers with hospitalization records in the DAD and emergency department (ED) records in the NACRS (2006‒2020). In a comparison of included workers with a HIN and those excluded without a HIN, excluded workers were significantly more likely to be female, older, and with an earlier year of entry into the ODSS (all *p* < 0.0001) than those who were included. Detailed information on the linkage process has been previously described (Jung et al., 2018).

Of the cohort of included workers linked to the DAD and NACRS (*n* = 1,973,312), a total of 283,677 workers were not eligible for follow-up in this analysis, due to being outside the working age range (15 to 65 years) during the follow-up period of 2006 to 2020 or death/emigration out of Ontario before the follow-up period. As a result, 1,689,635 workers were included in the analytical cohort for this analysis. Supplemental Figure 1 depicts the number of workers added to the ODSS by year from 1983 to 2019. The study protocol was approved by the University of Toronto Health Sciences Research Ethics Board (reference 39013).

### Outcomes

Two outcomes were examined in this study: opioid-related poisonings and opioid-related mental and behavioural disorders. Hospitalizations and ED visits for each outcome were identified separately in the DAD and NACRS, respectively, from 2006 to 2020 using diagnoses coded according to the International Classification of Diseases and Related Health Problems, 10th Revision, Canada (ICD-10-CA) (poisonings: T40.0–T40.4, T40.6; mental and behavioural disorders: F11.0–F11.9; see Supplemental Table 1 for a detailed list). These case definitions have been used previously (Special Advisory Committee on the Epidemic of Opioid Overdoses, 2020). Poisoning intent was identified using the following ICD-10-CA diagnostic codes: X42, accidental poisonings; X62, intentional poisonings; and Y12, unknown poisonings.

### Covariates

For each worker in the ODSS, information on occupation and industry was obtained from WSIB records at the time of claim. Occupation was coded using the 1971 Canadian Classification Dictionary of Occupation at the 4-digit level. Using these codes, occupations were classified into 22 division groups, the broadest level of classification. Similarly, industry was coded according to the 1970 and 1980 Canadian Standard Industrial Classification into 10 industry division groups.

In addition, worker age, sex, and postal code information were obtained from the RPDB. Postal code data were used to identify workers’ provincial public health units using Statistics Canada’s Postal Code Conversion File. Due to sparse data in some public health units, data from the 35 public health units were then grouped into seven health regions.

### Statistical analysis

Workers were followed for each opioid-related harm from 2006 or their first WSIB-accepted claim (whichever came last) to the date of emigration, death, age of 65 years, or the end of study follow-up (December 31, 2020). Within this follow-up period, person-years and the observed number of cases of each outcome were obtained from the ODSS, by calendar year, age (in 10-year age groups), sex, and health region. Incidence rates for each outcome in the Ontario general population over the same time period (2006 to 2020) were calculated using data obtained from ICES (formerly known as the Institute for Clinical Evaluative Sciences). Approximately 9.2 to 10.2 million Ontarians from the general population were included in each year of the analysis. Analyses were conducted separately in each database (DAD and NACRS).

Standardized incidence ratios (SIRs) and corresponding 95% confidence intervals (CIs) were calculated for the overall cohort, as well as for each occupation and industry at the division level. The SIR estimates the occurrence of opioid-related harms among workers in the ODSS relative to what might be expected if workers in the ODSS had the same opioid-related harm experience as the general Ontario population. The SIR was calculated as the ratio of the total number of observed cases of each opioid-related harm in the ODSS to the number of expected cases. The expected number of cases is the number of cases that would occur among workers in the ODSS if the incidence rate in the general population occurred in the ODSS. General population incidence rates were multiplied by person-years from the ODSS to calculate the expected number of cases. Occupation and industry groups with observed cell counts smaller than 6 are not presented. All SIRs were adjusted for sex, age, calendar year, and health region. In a subgroup analysis, analyses were repeated among males and females separately. Analyses were also replicated in two sets of sensitivity analyses: (1) limiting the cohort to workers with more recent claims (2006 to 2020) to align with the follow-up period; and (2) limiting cases of poisonings to those coded as accidental. SIRs were interpreted as statistically significant at the 5% significance level. All analyses were performed using SAS V.9.4.

## Results

### Characteristics of the ODSS cohort and general Ontario population in 2010

A total of 1.69 million workers in the ODSS cohort were included in this analysis. The sociodemographic characteristics of these workers and the general Ontario population in 2010 are presented in Table 1. Most workers in the ODSS (80.9%) were at least 35 years of age, male (66.3%), and located in the Central East and Central West health regions of Ontario (27.8% and 21.5%, respectively). By comparison, in the general population, two thirds were at least 35 years old, half the population was male, and most individuals were in the Central East, Central West, and Toronto health regions.

**Table 1:** Characteristics of the Occupational Disease Surveillance System cohort (*N* = 1,689,635) and the Ontario general population in 2010 (*N* = 9,377,402)

Characteristics	ODSS cohort (*N* = 1,689,635)	Ontario general population in 2010 (*N* = 9,377,402)
Age in 2010, *n* (%)		
15‒24 years	118,271 (7.0)	1,761,950 (18.8)
25‒34 years	205,222 (12.1)	1,751,374 (18.7)
35‒44 years	364,212 (21.6)	1,989,251 (21.2)
45‒54 years	531,334 (31.4)	2,155,366 (23.0)
55‒65 years	470,596 (27.9)	1,719,461 (18.3)
Sex, *n* (%)		
Male	1,120,120 (66.3)	4,663,221 (49.7)
Female	569,515 (33.7)	4,714,181 (50.3)
Ontario Health Region^a^, *n* (%)		
Central East	469,548 (27.8)	2,725,399 (29.1)
Central West	363,406 (21.5)	1,756,358 (18.7)
Eastern	209,233 (12.4)	1,230,880 (13.1)
North East	96,958 (5.7)	404,258 (4.3)
North West	38,104 (2.3)	173,883 (1.9)
South West	253,183 (15.0)	1,103,083 (11.8)
Toronto	222,521 (13.2)	1,983,105 (21.1)

*ODSS*, Occupational Disease Surveillance System

^a^Numbers do not add up to total due to missing values

### Opioid-related harms in the ODSS compared to the general population

In total, 11,674 ED visits and 4500 hospitalizations for opioid-related poisonings were observed in the ODSS from 2006 to 2020. Approximately half were accidental and involved other opioids (e.g., codeine, morphine, hydromorphone, oxycodone) (Supplemental Table 2). Compared to the general population, the risk of opioid-related poisonings among workers in the ODSS was elevated in both ED visits (SIR = 2.41, 95% CI = 2.37–2.45) and hospitalization records (SIR = 1.54, 95% CI = 1.50–1.59).

A total of 16,570 ED visits for opioid-related mental and behavioural disorders were observed in the ODSS and were significantly elevated compared to the general population (SIR = 1.86, 95% CI = 1.83–1.89). In hospitalization records, 3671 cases of opioid-related mental and behavioural disorders were observed in the ODSS (SIR = 1.42, 95% CI = 1.38–1.47). The most common diagnoses for mental and behavioural disorders identified in the ODSS worker cohort were withdrawal state, dependence syndrome, and harmful use (Supplemental Table 3).

### Opioid-related poisonings in the ODSS compared to the general population, by occupation

**Fig. 1:**
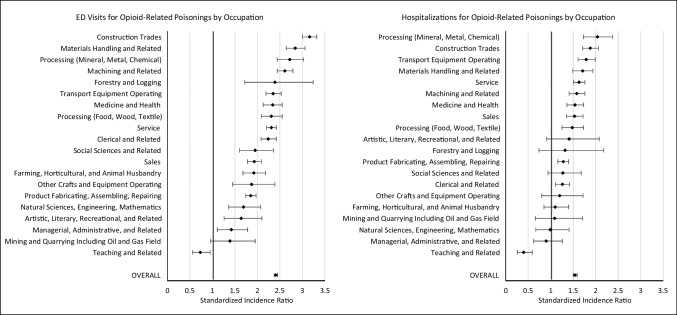
Standardized incidence ratios and corresponding 95% confidence intervals by occupation for opioid-related poisonings from 2006 to 2020. Abbreviation: ED, emergency department

Almost all occupational groups in the ODSS demonstrated elevated risks for opioid-related poisonings compared to the general population, as identified through ED visits (Fig. 1). Some of the highest SIRs were observed among workers in construction trades (SIR = 3.16, 95% CI = 3.00–3.32), materials handling (SIR = 2.84, 95% CI = 2.64–3.06), processing (mineral, metal, chemical) (SIR = 2.72, 95% CI = 2.44–3.03), and machining and related (SIR = 2.61, 95% CI = 2.44–2.79) occupations (see Supplemental Table 4 for detailed estimates).

Opioid-related poisonings identified through hospitalization data were also elevated across many occupations, particularly in processing (mineral, metal, chemical) (SIR = 2.04, 95% CI = 1.73–2.38), construction trades (SIR = 1.88, 95% CI = 1.71–2.06), and transport equipment operating (SIR = 1.79, 95% CI = 1.61–1.99) occupations (Fig. 1). Higher risks were also seen in materials handling and related occupations, service, machining and related occupations, medicine and health, sales, and processing (food, wood, textile) occupations (see Supplemental Table 4 for detailed estimates). Notably, teaching and related occupations were found to have a reduced risk of opioid-related poisonings compared to the general population, based on both ED and hospitalization data (SIR = 0.73, 95% CI = 0.56–0.95 and SIR = 0.40, 95% CI = 0.26–0.59, respectively).

### Opioid-related mental and behavioural disorders in the ODSS compared to the general population, by occupation

**Fig. 2:**
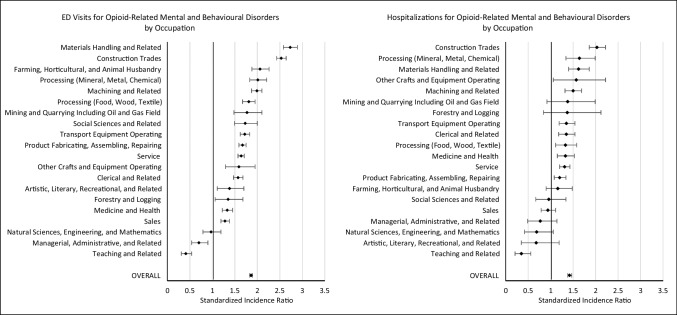
Standardized incidence ratios and corresponding 95% confidence intervals by occupation for opioid-related mental and behavioural disorders from 2006 to 2020. Abbreviation: ED, emergency department

Similar to poisonings, almost all occupational groups demonstrated elevated risks for opioid-related mental and behavioural disorders identified through ED visits (Fig. 2). Occupations with the highest risks included materials handling (SIR = 2.73, 95% CI = 2.58–2.89), construction trades (SIR = 2.53, 95% CI = 2.43–2.64), farming, horticultural, and animal husbandry (SIR = 2.06, 95% CI = 1.88–2.26), processing (mineral, metal, chemical) (SIR = 2.01, 95% CI = 1.83–2.21), and machining and related (SIR = 1.99, 95% CI = 1.87–2.10) occupations (see Supplemental Table 5 for detailed estimates). In both teaching (SIR = 0.41, 95% CI = 0.31–0.54) and managerial, administrative, and related (SIR = 0.70, 95% CI = 0.54–0.90) occupations, reduced risks of opioid-related mental and behavioural disorder ED visits compared to the general population were evident.

When examining opioid-related mental and behavioural disorders identified through hospitalization records, workers in construction trades (SIR = 2.03, 95% CI = 1.86–2.22), processing (mineral, metal, chemical) (SIR = 1.64, 95% CI = 1.34–1.99), and materials handling (SIR = 1.62, 95% CI = 1.40–1.86) occupations were found to have increased risks (Fig. 2). In general, risks were elevated across most occupation groups compared to the general population (see Supplemental Table 5 for detailed estimates). In contrast, workers in teaching and related occupations were found to have a reduced risk (SIR = 0.35, 95% CI = 0.21–0.56).

### Opioid-related poisonings and mental and behavioural disorders in the ODSS compared to the general population, by industry

When examining opioid-related poisonings identified through ED visits (Supplemental Table 6), elevated risks were observed across all industries at the division level. This included workers in construction (SIR = 3.19, 95% CI = 3.04–3.35), community, business, and personal service (SIR = 2.68, 95% CI = 2.59–2.78), forestry, fishing, and trapping (SIR = 2.56, 95% CI = 1.87–3.43), and manufacturing (SIR = 2.47, 95% CI = 2.38–2.56) industries. Similar findings were observed for opioid-related hospitalizations (Supplemental Table 6).

When examining opioid-related mental and behavioural disorders identified through ED visits (Supplemental Table 7), elevated risks were observed across all industries at the division level. This included construction (SIR = 2.65, 95% CI = 2.55–2.76), manufacturing (SIR = 2.04, 95% CI = 1.98–2.10), and community, business, and personal service (SIR = 1.92, 95% CI = 1.86–1.98) industries. Higher risks for hospitalizations were observed across several industries, including construction (SIR = 2.01, 95% CI = 1.83–2.20), transportation, communication, and other utilities (SIR = 1.55, 95% CI = 1.39–1.73), and manufacturing (SIR = 1.46, 95% CI = 1.37–1.55).

### Subgroup analyses stratified by sex

All analyses were repeated among males and females separately. Compared to among females in the general population, the risk of opioid-related poisonings among female workers in the ODSS was elevated in both ED visits (SIR = 2.83, 95% CI = 2.72–2.93) and hospitalization records (SIR = 1.60, 95% CI = 1.52–1.68). Results were similar for males (ED visits SIR = 2.30, 95% CI = 2.25–2.35; hospitalizations SIR = 1.52, 95% CI = 1.46–1.57). Likewise, the risk of opioid-related mental and behavioural disorders was elevated among both female workers in the ODSS (ED visits SIR = 1.73, 95% CI = 1.67–1.79; hospitalizations SIR = 1.33, 95% CI = 1.25–1.42) and their male counterparts (ED visits SIR = 1.90, 95% CI = 1.87–1.93; hospitalizations SIR = 1.46, 95% CI = 1.40–1.51) compared to among the general population. The pattern of findings by occupation and industry among both men and women was similar to those of the main analysis. However, in most cases, SIR estimates were higher among women than among men (see Supplemental Tables 4 to 7).

### Results of sensitivity analyses

When repeating analyses using a subset of workers with recent claims (2006 to 2020), the general pattern of findings was similar to that seen in the main analysis, although point estimates were often attenuated, and precision of estimates was reduced by the smaller sample size. When poisonings were limited to those coded as being accidental, the pattern of results was also similar. Details are available upon request.

## Discussion

Overall, this cohort of formerly injured workers demonstrated higher risks of both opioid-related poisonings and mental and behavioural disorders compared to the general Ontario population. Most occupational and industry groups were found to have increased risks of opioid-related harms. However, some of the largest and most consistent elevations in risk were observed among workers in construction, materials handling, processing (mineral, metal, chemical), and machining and related occupations, among other groups. Consistent decreases in risk were observed among workers in teaching and related occupations. The pattern of findings was generally robust in sex-stratified analyses and sensitivity analyses.

Findings from this study provide additional support to the limited but growing body of literature suggesting a role for work-related injuries as a risk factor for opioid-related harms (Martin et al., 2020; Cheng et al., 2013; Asfaw & Boden, 2020). Following a work-related injury, workers may face a number of different challenges that make them more vulnerable to experiencing opioid-related harms. Studies have shown these workers frequently face pressure to return to work and may lack suitable workplace accommodations, potentially making it more likely they will use opioids to manage residual pain (MacEachen et al., 2007; Shaw et al., 2020). Even long after injury, injured workers may continue to have disabling pain. A recent study of injured workers found that one in four workers reported severe pain intensity with substantial functional impairment at 18 months post-injury (Dobson et al., 2022). Yet, while opioids are commonly prescribed following an occupational injury to manage pain (Durand et al., 2019; Asfaw et al., 2022; Rosenman & Wang, 2022), opioid use has been shown to have a limited impact on pain and function among injured workers and, conversely, to be associated with long-term disability (Franklin et al., 2009; Tefera et al., 2023). Escalating doses and prolonged opioid use, as has been demonstrated in this subgroup of the labour market (Durand et al., 2019), also pose a risk for development of problematic use (Chou et al., 2015). In addition, injured workers frequently experience poor mental health that may further contribute to opioid use and related harms (Carnide et al., 2016). Finally, workers with work injuries may have difficulties returning to work (Sears et al., 2021), with intermittent interruptions in employment, long-term unemployment, and, in some cases, fully exiting the labour force, further placing them at risk of problematic use and opioid use disorders (Nagelhout et al., 2017). Taken together, findings from this and other studies underscore the importance of considering how occupational injuries may lead to opioid-related harms.

Results from this study are also consistent with the findings of previous studies identifying occupational patterns in opioid-related deaths among the general working population. Prior studies, primarily from the USA, have also found workers in jobs characterized by high levels of physical labour to be at greater risk of opioid-related mortality (Aram et al., 2020; Chalasani et al., 2020; Hawkins et al., 2019; Harduar Morano et al., 2018; Scagos et al., 2019; BC Coroners Service Death Review Panel, 2022; Gomes et al., 2022; Billock et al., 2023). In particular, in our study and others, workers in construction occupations have consistently ranked as having some of the highest rates of opioid-related deaths. For instance, Hawkins et al. found the rate of opioid-related poisoning deaths among construction workers in Massachusetts to be 124.9 per 100,000 workers (95% CI = 108.8–141.0) and significantly higher than workers in all other occupations (rate ratio 7.75, 95% CI = 7.24–8.30) (Hawkins et al., 2019). In our study, elevated risks of opioid-related harms were also observed among workers in occupations in medicine and health, sales, and service. While less consistently noted, some previous studies have also found higher rates of opioid use and related deaths in these occupations, particularly among food preparation service, personal care, and health care support occupations (Aram et al., 2020; Hawkins et al., 2019; Harduar Morano et al., 2018; Chalasani et al., 2020; Billock et al., 2023). In addition to higher underlying risks of work injuries in many of these occupations, job insecurity, precarious employment, financial instability, psychosocial work environment factors, and substance use workplace norms and availability may also be potential contributing factors, though few studies have specifically examined the contribution of these factors to the development of opioid-related harms among workers (Hawkins et al., 2021; Shaw et al., 2020).

Interestingly, teaching and related occupations were consistently shown to have decreased risks of opioid-related harms in our analysis. Some data from workers in the general working population also suggest workers in education occupations to be at reduced risk of opioid-related deaths compared to all other occupations (Hawkins et al., 2019; Harduar Morano et al., 2018), although rates of overall substance use in the USA have been reported to be increasing over time among those in the educational services industry (Bush & Lipari, 2013). Further research is needed to explore the reasons behind these findings.

While the overall pattern of findings was generally consistent among both males and females, point estimates of risk among females tended to exceed those of males for both sets of outcomes and data sources. The reasons for these differences are unclear but may point to differential impacts of work-related injuries on females versus males. For example, females have been shown in some studies to be more likely to experience poor mental health after injury and worse recovery (Jones et al., 2021; Stock & Nicolakakis, 2016), although this is not seen in other studies (Macpherson et al., 2019; Bultmann et al., 2007). More research is needed to confirm and better understand sex differences in risk of opioid-related harms after injury.

Results provide further support to the hypothesized role of work-related injuries as a determinant of opioid-related harms. As such, findings reinforce previous recommendations calling for action in the primary prevention of occupational injuries and pain, including improving ergonomics and reducing physical hazards in the workplace, as well as workplace psychosocial factors that could increase the risk of injury (Shaw et al., 2020). Following work injury, secondary prevention strategies aimed at the prevention of work disability and long-term opioid use are also important, including judicious prescribing, access to effective non-opioid treatments for pain, mental health supports, availability of sick leave, and workplace practices that assist workers in returning to meaningful work (e.g., through work accommodations). Educational campaigns that aim to increase awareness among workers about injury risk factors and the potential risks of opioid use for pain may also be important. Finally, workplace policies that treat substance use as a health issue may also help to create a supportive workplace culture that reduces stigma and encourages disclosure if workers develop an opioid use disorder. It is clear there is an opportunity for the workplace to act as a unique point of public health intervention. Future research using both quantitative and qualitative methodology should be conducted to more explicitly identify how workplace injuries lead to opioid-related harms, including investigating the impact of the downstream effects of work-related injuries and workplace characteristics on the pathway to harm.

### Strengths and limitations of the study

This study has several strengths. To our knowledge, this is one of the first studies to empirically examine the role of work-related injury in opioid-related harms, as well as to examine variation in this relationship by occupation and industry. These findings also expand upon the existing literature, which has mainly focused on opioid-related mortality, to include cases appearing in emergency department and hospitalization records, allowing for a more comprehensive capture of opioid-related harms. In addition, this analysis used data from a large cohort of workers, allowing for greater precision and statistical power.

There are also some limitations. Cases of opioid-related poisonings and mental and behavioural disorders occurring in the community that did not present to hospital will not have been captured in our data, likely leading to non-differential misclassification of our outcome. Only a limited set of variables (age, sex, calendar year, region) were controlled for in the analysis, as data on other important potential confounders (e.g., race, trauma history, socioeconomic status) were not available. Thus, residual confounding may also be present, potentially biasing our estimates away from the null. Data on occupation and industry were obtained at the time of the workers’ compensation claim and some non-differential misclassification in these measures is possible if workers changed jobs, potentially leading to an attenuation of the point estimates. Workers with work-related injuries who did not file a claim with the workers’ compensation authority in Ontario would not have been captured, limiting the generalizability of our findings. Nevertheless, the ODSS cohort is one of the only worker cohorts in Canada that contains data on occupational history. Finally, as previously described, records from approximately 17% of the original cohort of 2.38 million workers gathered to create the ODSS were excluded from the analysis, due to their lack of a unique identifier required to link to hospitalization and ED records. The direction of potential selection bias introduced because of this exclusion is difficult to predict. It is possible that our SIR estimates were overestimated, due to the fact that those who were included were more likely to be male and younger, groups disproportionately affected by this crisis (Federal, provincial, and territorial Special Advisory Committee on the Epidemic of Opioid Overdoses, 2023). However, we lacked data on other characteristics to fully appraise the potential for and direction of selection bias in our analysis.

## Conclusion

This cohort of formerly injured workers demonstrated elevated risks of hospital encounters (emergency department visits and hospitalizations) for both opioid-related poisonings and opioid-related mental and behavioural disorders compared to the general population. Findings emphasize the role of work-related injury as a preventable risk factor for opioid-related harms.

## Contributions to knowledge

What does this study add to existing knowledge?

- While the role of work-related injuries as a determinant of opioid-related harms has been hypothesized, few studies have empirically examined this issue, nor assessed whether risks differ by occupation.
- In this study, a cohort of formerly injured workers demonstrated higher risks of both opioid-related poisonings and mental and behavioural disorders compared to the general Ontario population.
- Most occupational and industry groups were found to have increased risks of opioid-related harms, although certain occupations appear to be at particularly high risk, namely construction, materials handling, processing (mineral, metal, chemical), and machining and related occupations.

What are the key implications for public health interventions, practice, or policy?

- Findings support the hypothesis that work-related injuries are a preventable risk factor for opioid-related harms.
- Strategies aimed at prevention of occupational injuries are warranted (e.g., improving ergonomics, reducing physical and psychosocial hazards in the workplace, educating workers on injury risk factors), as well as those targeting prevention of work disability and long-term opioid use (e.g., judicious prescribing, access to effective non-opioid treatments for pain, mental health supports, availability of sick leave, work accommodations, educating workers on risks of opioid use). Supportive workplace policies may also help encourage disclosure if workers develop an opioid use disorder.

## Supplementary Information

Below is the link to the electronic supplementary material.

Supplementary file1 (PDF 429 KB)

Supplementary file2 (PDF 78.5 KB)

## Data Availability

Data are available upon reasonable request. Data may be obtained from a third party and are not publicly available.

## Code availability

The SAS codes used in the analyses are available upon reasonable request to the corresponding author.
