# Supplementary material for: Occupational patterns of opioid-related harms comparing a cohort of formerly injured workers to the general population in Ontario, Canada
Source: Can J Public Health. 2024 Apr 24;115(6):851–61. doi: 10.17269/s41997-024-00882-w (PMC11638427; doi:10.17269/s41997-024-00882-w)
Supplement: Supplementary file 1 — Supplementary file1 (PDF 429 KB) [file 41997_2024_882_MOESM1_ESM.pdf]

SUPPLEMENTAL TABLES

Occupational Patterns of Opioid-Related Harms Comparing a Cohort of Formerly Injured Workers to the General Population in Ontario, Canada

Carnide N, Feng G, Song C, Demers PA, MacLeod JS, Sritharan J.

Supplemental Table 1. ICD-10-CA diagnostic codes used to identify opioid-related harms in hospitalization and emergency department records

Outcome	ICD-10-CA Code
**Opioid-Related Poisonings**	
Opioid Poisoning	T40.0-T40.4, T40.6
Poisoning by opium	T40.0
Poisoning by heroin	T40.1
Poisoning by other opioids	T40.2
Poisoning by codeine and derivatives	T40.20[1]
Poisoning by morphine	T40.21[1]
Poisoning by hydromorphone	T40.22[1]
Poisoning by oxycodone	T40.23[1]
Poisoning by other opioids, not elsewhere classified	T40.28[1]
Poisoning by methadone	T40.3
Poisoning by other synthetic narcotics	T40.4
Poisoning by fentanyl and derivatives	T40.40[1]
Poisoning by tramadol	T40.41[1]
Poisoning by other synthetic narcotics, not elsewhere classified	T40.48[1]
Poisoning by unspecified and other narcotics	T40.6
**Opioid-Related Mental and Behavioural Disorders**	
Mental and behavioural disorders due to use of opioids	F11.0-F11.9
Mental and behavioural disorders due to use of opioids, acute intoxication	F11.0
Mental and behavioural disorders due to use of opioids, harmful use	F11.1
Mental and behavioural disorders due to use of opioids, dependence syndrome	F11.2
Mental and behavioural disorders due to use of opioids, withdrawal state	F11.3
Mental and behavioural disorders due to use of opioids, withdrawal state with delirium	F11.4
Mental and behavioural disorders due to use of opioids, psychotic disorder	F11.5
Mental and behavioural disorders due to use of opioids, amnesic syndrome	F11.6
Mental and behavioural disorders due to use of opioids, residual and late-onset psychiatric disorder	F11.7
Mental and behavioural disorders due to use of opioids, other mental and behavioural disorders	F11.8
Mental and behavioural disorders due to use of opioids, unspecified mental and behavioural disorder	F11.9

Abbreviations: ICD-10-CA = International Classification of Diseases and Related Health Problems, 10th Revision, Canada
[1] These diagnostic codes were introduced as of April 1, 2018.

Supplemental Table 2. Opioid-related poisoning events by intent of poisoning and type of opioid involved in the poisoning event in the ODSS worker cohort (2006-2020)

	Emergency Department Visits for Poisonings (N=11,674)		Hospitalizations for Poisonings (N=4,500)	
	N	%	N	%
**Intent of Poisoning[1]**				
Accidental	6,304	54.0	2,131	47.4
Intentional	2,922	25.0	1,485	33.0
Unknown	1,948	16.7	656	14.6
Missing	514	4.4	237	5.3
**Opioid(s) involved in poisoning[1,2]**				
Opium	49	0.4	23	0.5
Heroin	1,264	10.8	149	3.3
Other opioids[3]	6,029	51.6	2,700	60.0
Methadone	964	8.3	535	11.9
Other synthetic narcotics[3]	2,446	21.0	465	10.3
Opiates, not elsewhere classified	1,473	12.6	852	18.9

[1] Identified using diagnostic codes set out in the International Statistical Classification of Diseases and Related Health Problems, Tenth Revision, Canada (ICD-10-CA).

[2] Multiple opioids may be present in a single poisoning event. Therefore, these categories are not mutually exclusive and, as a result, frequencies exceed totals and the sum of the percentages is greater than 100%.

[3] Other opioids include codeine and derivatives, morphine, hydromorphone, and oxycodone. Other synthetic narcotics include fentanyl and derivatives and tramadol.

Supplemental Table 3. Opioid-related mental and behavioural disorder events by type of disorder in the ODSS worker cohort (2006-2020)

Type of Mental and Behavioural Disorder[1,2]	Emergency Department Visits for Mental and Behavioural Disorders (N=16,570)[3]		Hospitalizations for Mental and Behavioural Disorders (N=3,671)[3]	
	N	%	N	%
Acute intoxication	770	4.6	294	8.0
Amnesic syndrome	<6		<6	
Dependence syndrome	4,361	26.3	1,290	35.1
Harmful use	4,129	24.9	841	22.9
Other mental and behavioural disorder	85	0.5	22	0.6
Psychotic disorder	143	0.9	49	1.3
Residual and late-onset psychotic disorder	<6		<6	
Unspecified mental and behavioural disorder	228	1.4	66	1.8
Withdrawal state	7,324	44.2	1,208	32.9
Withdrawal state with delirium	60	0.4	138	3.8

[1] Identified using diagnostic codes set out in the International Statistical Classification of Diseases and Related Health Problems, Tenth Revision, Canada (ICD-10-CA).

[2] Categories are not mutually exclusive and, as a result, frequencies exceed totals and the sum of the percentages is greater than 100%.

[3] Where cases are less than six, data are not reported.

Supplemental Table 4. Standardized incidence ratios (SIRs) and corresponding 95% confidence intervals (CIs) by occupation for opioid-related poisonings, overall and stratified by sex

		Emergency Department Visits			Hospitalizations		
**Occupation (Division Level)**	**Sex**	**Exp**	**Obs**	**SIR[1,2,3] (95% CI)**	**Exp**	**Obs**	**SIR[1,2,3] (95% CI)**
Managerial, Administrative, and Related	Both	52.30	74	**1.42 (1.11-1.78)**	37.85	34	0.90 (0.62-1.26)
	Females	22.04	42	**1.91 (1.37-2.58)**	21.72	20	0.92 (0.56-1.42)
	Males	30.25	32	1.06 (0.72-1.49)	16.13	14	0.87 (0.47-1.46)
Natural Sciences, Engineering, Mathematics	Both	53.93	91	**1.69 (1.36-2.07)**	31.26	31	0.99 (0.67-1.41)
	Females	7.48	21	**2.81 (1.74-4.29)**	6.72	11	1.64 (0.82-2.93)
	Males	46.44	70	**1.51 (1.17-1.90)**	24.54	20	0.82 (0.50-1.26)
Social Sciences and Related	Both	54.26	106	**1.95 (1.60-2.36)**	38.55	49	1.27 (0.94-1.68)
	Females	35.43	77	**2.17 (1.71-2.72)**	29.62	39	1.32 (0.94-1.80)
	Males	18.83	29	**1.54 (1.03-2.21)**	8.93	10	1.12 (0.54-2.06)
Religion	Both	-	<6	-	-	<6	-
	Females						
	Males						
Teaching and Related	Both	78.97	58	***0.73 (0.56-0.95)***	63.01	25	***0.40 (0.26-0.59)***
	Females	54.83	45	0.82 (0.60-1.10)			
	Males	24.14	13	***0.54 (0.29-0.92)***		<6	-
Medicine and Health	Both	207.18	484	**2.34 (2.13-2.55)**	172.64	265	**1.54 (1.36-1.73)**
	Females	158.06	414	**2.62 (2.37-2.88)**	149.60	229	**1.53 (1.34-1.74)**
	Males	49.13	70	**1.42 (1.11-1.80)**	23.03	36	**1.56 (1.09-2.16)**
Artistic, Literary, Recreational, and Related	Both	37.83	62	**1.64 (1.26-2.10)**	17.78	25	1.41 (0.91-2.08)
	Females	12.76	27	**2.12 (1.39-3.08)**	7.60	6	0.79 (0.29-1.72)
	Males	25.07	35	1.40 (0.97-1.94)	10.18	19	**1.87 (1.12-2.92)**
Clerical and Related	Both	304.19	682	**2.24 (2.08-2.42)**	209.39	264	**1.26 (1.11-1.42)**
	Females	112.31	332	**2.96 (2.65-3.29)**	111.11	145	**1.31 (1.10-1.54)**
	Males	191.88	350	**1.82 (1.64-2.03)**	98.29	119	**1.21 (1.00-1.45)**
Sales	Both	320.65	618	**1.93 (1.78-2.09)**	171.67	262	**1.53 (1.35-1.72)**
	Females	105.62	282	**2.67 (2.37-3.00)**	84.33	152	**1.80 (1.53-2.11)**
	Males	215.03	336	**1.56 (1.40-1.74)**	87.34	110	**1.26 (1.04-1.52)**
Service	Both	698.14	1,612	**2.31 (2.20-2.42)**	406.06	661	**1.63 (1.51-1.76)**
	Females	226.25	704	**3.11 (2.89-3.35)**	188.50	353	**1.87 (1.68-2.08)**

Occupation (Division Level)	Sex	Emergency Department Visits			Hospitalizations		
		Exp	Obs	SIR[1,2,3] (95% CI)	Exp	Obs	SIR[1,2,3] (95% CI)
	Males	471.89	908	**1.92 (1.80-2.05)**	217.56	308	**1.42 (1.26-1.58)**
Farming, Horticultural, and Animal Husbandry	Both	125.08	240	**1.92 (1.68-2.18)**	59.26	65	1.10 (0.85-1.40)
	Females	16.76	45	**2.68 (1.96-3.59)**	11.92	12	1.01 (0.52-1.76)
	Males	108.32	195	**1.80 (1.56-2.07)**	47.34	53	1.12 (0.84-1.46)
Fishing, Hunting, Trapping, and Related	Both	-	<6	–	-	<6	–
	Females						
	Males						
Forestry and Logging	Both	17.15	41	**2.39 (1.72-3.24)**	11.36	15	1.32 (0.74-2.18)
	Females	-	<6	–	-	<6	–
	Males						
Mining and Quarrying (including oil and gas field)	Both	23.78	33	1.39 (0.96-1.95)	17.38	19	1.09 (0.66-1.71)
	Females	-	<6	–	-	<6	–
	Males						
Processing (mineral, metal, chemical)	Both	123.43	336	**2.72 (2.44-3.03)**	77.54	158	**2.04 (1.73-2.38)**
	Females	14.99	46	**3.07 (2.25-4.09)**	15.67	30	**1.91 (1.29-2.73)**
	Males	108.44	290	**2.67 (2.38-3.00)**	61.87	128	**2.07 (1.73-2.46)**
Processing (food, wood, textile)	Both	169.34	391	**2.31 (2.09-2.55)**	105.06	155	**1.48 (1.25-1.73)**
	Females	33.65	88	**2.62 (2.10-3.22)**	32.78	51	**1.56 (1.16-2.05)**
	Males	135.69	303	**2.23 (1.99-2.50)**	72.28	104	**1.44 (1.18-1.74)**
Machining and Related	Both	327.13	855	**2.61 (2.44-2.79)**	201.44	318	**1.58 (1.41-1.76)**
	Females	17.75	71	**4.00 (3.12-5.05)**	19.64	36	**1.83 (1.28-2.54)**
	Males	309.38	784	**2.53 (2.36-2.72)**	181.80	282	**1.55 (1.38-1.74)**
Product Fabricating, Assembling, Repairing	Both	552.56	1,022	**1.85 (1.74-1.97)**	350.52	447	**1.28 (1.16-1.40)**
	Females	56.21	170	**3.02 (2.59-3.51)**	62.64	119	**1.90 (1.57-2.27)**
	Males	496.35	852	**1.72 (1.60-1.84)**	287.87	328	**1.14 (1.02-1.27)**
Construction Trades	Both	485.18	1,533	**3.16 (3.00-3.32)**	249.39	469	**1.88 (1.71-2.06)**
	Females	5.97	22	**3.69 (2.31-5.58)**	4.84	13	**2.69 (1.43-4.59)**
	Males	479.22	1,511	**3.15 (3.00-3.32)**	244.55	456	**1.86 (1.70-2.04)**
Transport Equipment Operating	Both	318.24	748	**2.35 (2.19-2.53)**	194.02	348	**1.79 (1.61-1.99)**
	Females	16.95	69	**4.07 (3.17-5.15)**	16.68	39	**2.34 (1.66-3.20)**

Occupation (Division Level)	Sex	Emergency Department Visits			Hospitalizations		
		Exp	Obs	SIR[1,2,3] (95% CI)	Exp	Obs	SIR[1,2,3] (95% CI)
	Males	301.29	679	**2.25 (2.09-2.43)**	177.34	309	**1.74 (1.55-1.95)**
Materials Handling and Related	Both	252.78	719	**2.84 (2.64-3.06)**	137.82	235	**1.71 (1.49-1.94)**
	Females	24.13	83	**3.44 (2.74-4.26)**	23.94	45	**1.88 (1.37-2.52)**
	Males	228.65	636	**2.78 (2.57-3.01)**	113.88	190	**1.67 (1.44-1.92)**
Other Crafts and Equipment Operating	Both	35.20	66	**1.87 (1.45-2.39)**	24.20	29	1.20 (0.80-1.72)
	Females	4.93	9	1.82 (0.83-3.46)	5.41	7	1.29 (0.52-2.67)
	Males	30.27	57	**1.88 (1.43-2.44)**	18.79	22	1.17 (0.73-1.77)
Not Elsewhere Classified	Both	382.46	1,234	**2.32 (3.05-3.41)**	200.18	384	**1.92 (1.73-2.12)**
	Females	39.81	156	**3.92 (3.33-4.58)**	36.49	75	**2.06 (1.62-2.58)**
	Males	342.65	1,078	**3.15 (2.96-3.34)**	163.70	309	**1.89 (1.68-2.11)**

Abbreviations: CI = confidence interval; Exp = expected cases; Obs = observed cases; SIR = standardized incidence ratio

[1] Overall findings adjusted for sex, age, calendar year, and health region. Sex-specific findings adjusted for age, calendar year, and health region.

[2] Bolded values denote statistically significant SIRs above 1. Italicized bolded values denote statistically significant SIRs below 1.

[3] SIRs are not reported where the observed number of cases are less than 6.

Supplemental Table 5. Standardized incidence ratios (SIRs) and corresponding 95% confidence intervals (CI) by occupation for opioid-related mental and behavioural disorders, overall and stratified by sex

		Emergency Department Visits			Hospitalizations		
**Occupation (Division Level)**	**Sex**	**Exp**	**Obs**	**SIR[1,2,3] (95% CI)**	**Exp**	**Obs**	**SIR[1,2,3] (95% CI)**
Managerial, Administrative, and Related	Both	85.37	60	***0.70 (0.54-0.90)***	31.35	24	0.77 (0.49-1.14)
	Females	37.35	24	***0.64 (0.41-0.96)***	16.44	10	0.61 (0.29-1.12)
	Males	48.02	36	*0.75 (0.53-1.04)*	14.91	14	0.94 (0.51-1.58)
Natural Sciences, Engineering, Mathematics	Both	99.57	97	0.97 (0.79-1.19)	29.06	20	0.69 (0.42-1.06)
	Females	14.20	13	0.92 (0.49-1.57)	-	<6	–
	Males	85.37	84	0.98 (0.78-1.22)			
Social Sciences and Related	Both	105.93	183	**1.73 (1.49-2.00)**	36.30	35	0.96 (0.67-1.34)
	Females	70.90	147	**2.07 (1.75-2.44)**	27.25	26	0.95 (0.62-1.40)
	Males	35.03	36	1.03 (0.72-1.42)	9.05	9	0.99 (0.45-1.89)
Religion	Both	-	<6	–	-	<6	–
	Females						
	Males						
Teaching and Related	Both	130.72	54	***0.41 (0.31-0.54)***	51.15	18	***0.35 (0.21-0.56)***
	Females	91.64	43	***0.47 (0.34-0.63)***			
	Males	39.08	11	***0.28 (0.14-0.50)***	-	<6	–
Medicine and Health	Both	390.18	519	**1.33 (1.22-1.45)**	145.57	194	**1.33 (1.15-1.53)**
	Females	302.64	449	**1.48 (1.35-1.63)**	122.40	165	**1.35 (1.15-1.57)**
	Males	87.54	70	0.80 (0.62-1.01)	23.17	29	1.25 (0.84-1.80)
Artistic, Literary, Recreational, and Related	Both	65.75	91	**1.38 (1.11-1.70)**	17.56	12	0.68 (0.35-1.19)
	Females	23.36	27	1.16 (0.76-1.68)	-	<6	–
	Males	42.40	64	**1.51 (1.16-1.93)**			
Clerical and Related	Both	530.49	833	**1.57 (1.47-1.68)**	171.47	232	**1.35 (1.18-1.54)**
	Females	205.99	342	**1.66 (1.49-1.85)**	82.99	110	**1.33 (1.09-1.60)**
	Males	324.50	491	**1.51 (1.38-1.65)**	88.48	122	**1.38 (1.15-1.65)**
Sales	Both	571.12	732	**1.28 (1.19-1.38)**	154.39	145	0.94 (0.79-1.11)
	Females	197.60	268	**1.36 (1.20-1.53)**	71.98	78	1.08 (0.86-1.35)
	Males	373.51	464	**1.24 (1.13-1.36)**	82.41	67	0.81 (0.63-1.03)
Service	Both	1,294.18	2,125	**1.64 (1.57-1.71)**	374.56	491	**1.31 (1.20-1.43)**
	Females	444.41	854	**1.92 (1.79-2.06)**	164.65	266	**1.62 (1.43-1.82)**

Occupation (Division Level)	Sex	Emergency Department Visits			Hospitalizations		
		Exp	Obs	SIR[1,2,3] (95% CI)	Exp	Obs	SIR[1,2,3] (95% CI)
	Males	849.77	1,271	**1.50 (1.41-1.58)**	209.91	225	1.07 (0.94-1.22)
Farming, Horticultural, and Animal Husbandry	Both	224.79	464	**2.06 (1.88-2.26)**	55.04	64	1.16 (0.90-1.48)
	Females	32.06	65	**2.03 (1.56-2.58)**	10.72	12	1.12 (0.58-1.96)
	Males	192.73	399	**2.07 (1.87-2.28)**	44.33	52	1.17 (0.88-1.54)
Fishing, Hunting, Trapping, and Related	Both	2.42	7	**2.89 (1.16-5.96)**	-	<6	–
	Females	-	<6	–			
	Males						
Forestry and Logging	Both	57.94	78	**1.35 (1.06-1.68)**	14.58	20	1.37 (0.84-2.12)
	Females	-	<6	–	-	<6	–
	Males						
Mining and Quarrying (including oil and gas field)	Both	76.25	135	**1.77 (1.48-2.10)**	20.30	28	1.38 (0.92-1.99)
	Females	0.49	12	**24.76 (12.78-43.26)**	-	<6	–
	Males	75.76	123	**1.62 (1.35-1.94)**			
Processing (mineral, metal, chemical)	Both	220.20	443	**2.01 (1.83-2.21)**	63.91	105	**1.64 (1.34-1.99)**
	Females	25.02	58	**2.32 (1.76-3.00)**	9.93	18	**1.81 (1.07-2.86)**
	Males	195.18	385	**1.97 (1.78-2.18)**	53.98	87	**1.61 (1.29-1.99)**
Processing (food, wood, textile)	Both	340.59	615	**1.81 (1.67-1.95)**	94.03	125	**1.33 (1.11-1.58)**
	Females	60.45	92	**1.52 (1.23-1.87)**	23.53	13	***0.55 (0.29-0.94)***
	Males	280.15	523	**1.87 (1.71-2.03)**	70.50	112	**1.59 (1.31-1.91)**
Machining and Related	Both	596.73	1,185	**1.99 (1.87-2.10)**	169.85	254	**1.50 (1.32-1.69)**
	Females	31.09	82	**2.64 (2.10-3.27)**	12.32	27	**2.19 (1.44-3.19)**
	Males	565.64	1,103	**1.95 (1.84-2.07)**	157.53	227	**1.44 (1.26-1.64)**
Product Fabricating, Assembling, Repairing	Both	999.96	1,668	**1.67 (1.59-1.75)**	295.86	356	**1.20 (1.08-1.34)**
	Females	93.82	202	**2.15 (1.87-2.47)**	38.80	54	**1.39 (1.05-1.82)**
	Males	906.13	1,466	**1.62 (1.54-1.70)**	257.06	302	**1.17 (1.05-1.32)**
Construction Trades	Both	890.40	2,254	**2.53 (2.43-2.64)**	237.00	482	**2.03 (1.86-2.22)**
	Females	11.97	40	**3.34 (2.39-4.55)**	4.38	9	2.05 (0.94-3.90)
	Males	878.43	2,214	**2.52 (2.42-2.63)**	232.61	473	**2.03 (1.85-2.23)**
Transport Equipment Operating	Both	593.81	1,021	**1.72 (1.62-1.83)**	177.27	240	**1.35 (1.19-1.54)**

Occupation (Division Level)	Sex	Emergency Department Visits			Hospitalizations		
		Exp	Obs	SIR[1,2,3] (95% CI)	Exp	Obs	SIR[1,2,3] (95% CI)
	Females	31.09	54	**1.74 (1.30-2.27)**	12.74	25	**1.96 (1.27-2.90)**
	Males	562.72	967	**1.72 (1.61-1.83)**	164.53	215	**1.31 (1.14-1.49)**
Materials Handling and Related	Both	434.34	1,186	**2.73 (2.58-2.89)**	118.02	191	**1.62 (1.40-1.86)**
	Females	38.54	79	**2.05 (1.62-2.55)**	15.73	18	1.14 (0.68-1.81)
	Males	395.80	1,107	**2.80 (2.63-2.97)**	102.29	173	**1.69 (1.45-1.96)**
Other Crafts and Equipment Operating	Both	59.62	95	**1.59 (1.29-1.95)**	19.80	31	**1.57 (1.06-2.22)**
	Females	8.17	10	1.22 (0.59-2.25)	-	<6	–
	Males	51.45	85	**1.65 (1.32-2.04)**			
Not Elsewhere Classified	Both	702.39	1,819	**2.59 (2.47-2.71)**	179.02	372	**2.08 (1.87-2.30)**
	Females	70.09	200	**2.85 (2.47-3.28)**	26.43	58	**2.19 (1.67-2.84)**
	Males	632.30	1,619	**2.56 (2.44-2.69)**	152.59	314	**2.06 (1.84-2.30)**

Abbreviations: CI = confidence interval; Exp = expected cases; Obs = observed cases; SIR = standardized incidence ratio

[1] Overall findings adjusted for sex, age, calendar year, and health region. Sex-specific findings adjusted for age, calendar year, and health region.

[2] Bolded values denote statistically significant SIRs above 1. Italicized bolded values denote statistically significant SIRs below 1.

[3] SIRs are not reported where the observed number of cases are less than 6.

Supplemental Table 6. Standardized incidence ratios (SIRs) and corresponding 95% confidence intervals (CI) by industry for opioid-related poisonings, overall and stratified by sex

		Emergency Department Visits			Hospitalizations		
**Industry (Division Level)**	**Sex**	**Exp**	**Obs**	**SIR[1,2] (95% CI)**	**Exp**	**Obs**	**SIR[1,2] (95% CI)**
Agriculture	Both	83.89	121	**1.44 (1.20-1.72)**	43.69	42	0.96 (0.69-1.30)
	Females	11.47	27	**2.35 (1.55-3.42)**	9.69	7	0.72 (0.29-1.49)
	Males	72.42	94	**1.30 (1.05-1.59)**	34.00	35	1.03 (0.72-1.43)
Forestry, Fishing, and Trapping	Both	17.55	45	**2.56 (1.87-3.43)**	12.81	23	**1.80 (1.14-2.69)**
	Females	-	<6	-	-	<6	**-**
	Males						
Finance, Insurance, and Real Estate	Both	34.80	79	**2.27 (1.80-2.83)**	22.83	28	1.23 (0.81-1.77)
	Females	8.82	22	**2.49 (1.56-3.78)**	-	<6	**-**
	Males	25.98	57	**2.19 (1.66-2.84)**			
Mines (including milling), Quarries, Oil Wells	Both	41.25	66	**1.60 (1.24-2.04)**	31.38	45	**1.43 (1.05-1.92)**
	Females	-	<6	-	-	<6	**-**
	Males						
Manufacturing	Both	1,211.08	2,992	**2.47 (2.38-2.56)**	796.07	1,241	**1.56 (1.47-1.65)**
	Females	157.71	497	**3.15 (2.88-3.44)**	171.56	304	**1.77 (1.58-1.98)**
	Males	1,053.37	2,495	**2.37 (2.28-2.46)**	624.51	937	**1.50 (1.41-1.60)**
Construction	Both	513.17	1,636	**3.19 (3.04-3.35)**	254.53	508	**2.00 (1.83-2.18)**
	Females	13.07	47	**3.59 (2.64-4.78)**	11.57	21	**1.82 (1.12-2.78)**
	Males	500.09	1,589	**3.18 (3.02-3.34)**	242.96	487	**2.00 (1.83-2.19)**
Trade	Both	962.79	1,999	**2.08 (1.99-2.17)**	527.11	735	**1.39 (1.30-1.50)**
	Females	181.73	500	**2.75 (2.52-3.00)**	155.52	236	**1.52 (1.33-1.72)**
	Males	781.06	1,499	**1.92 (1.82-2.02)**	371.58	499	**1.34 (1.23-1.47)**
Transportation, Communication, Other Utilities	Both	384.08	862	**2.24 (2.10-2.40)**	233.83	351	**1.50 (1.35-1.67)**
	Females	42.55	135	**3.17 (2.66-3.76)**	41.10	60	**1.46 (1.11-1.88)**
	Males	341.53	727	**2.13 (1.98-2.29)**	192.74	291	**1.51 (1.34-1.69)**
Community, Business, and Personal Service	Both	1,149.31	3,082	**2.68 (2.59-2.78)**	718.18	1,191	**1.66 (1.57-1.76)**
	Females	473.97	1,371	**2.89 (2.74-3.05)**	416.69	702	**1.68 (1.56-1.81)**
	Males	675.34	1,711	**2.53 (2.41-2.66)**	301.49	489	**1.62 (1.48-1.77)**
Public Administration and Defence	Both	328.33	513	**1.56 (1.43-1.70)**	225.22	250	1.11 (0.98-1.26)
	Females	85.71	193	**2.25 (1.95-2.59)**	82.62	106	**1.28 (1.05-1.55)**

Industry (Division Level)	Sex	Emergency Department Visits			Hospitalizations		
		Exp	Obs	SIR[1,2] (95% CI)	Exp	Obs	SIR[1,2] (95% CI)
	Males	242.62	320	**1.32 (1.18-1.47)**	142.60	144	1.01 (0.85-1.19)

Abbreviations: CI = confidence interval; Exp = expected cases; Obs = observed cases; SIR = standardized incidence ratio

[1] Overall findings adjusted for sex, age, calendar year, and health region. Sex-specific findings adjusted for age, calendar year, and health region.

[2] Bolded values denote statistically significant SIRs above 1. Italicized bolded values denote statistically significant SIRs below 1.

Supplemental Table 7. Standardized incidence ratios (SIRs) and corresponding 95% confidence intervals (CI) by industry for opioid-related mental and behavioural disorders, overall and stratified by sex

		Emergency Department Visits			Hospitalizations		
**Industry (Division Level)**	**Sex**	**Exp**	**Obs**	**SIR[1,2] (95% CI)**	**Exp**	**Obs**	**SIR[1,2] (95% CI)**
Agriculture	Both	156.88	215	**1.37 (1.19-1.57)**	39.28	34	0.87 (0.60-1.21)
	Females	21.68	30	1.38 (0.93-1.98)	-	<6	-
	Males	135.21	185	**1.37 (1.18-1.58)**			
Forestry, Fishing, and Trapping	Both	66.74	101	**1.51 (1.23-1.84)**	16.44	13	0.79 (0.42-1.35)
	Females	-	<6	-	-	<6	-
	Males						
Finance, Insurance, and Real Estate	Both	64.24	119	**1.85 (1.53-2.22)**	20.72	17	0.82 (0.48-1.31)
	Females	15.40	27	**1.75 (1.16-2.55)**	-	<6	-
	Males	48.85	92	**1.88 (1.52-2.31)**			
Mines (including milling), Quarries, Oil Wells	Both	128.30	234	**1.82 (1.60-2.07)**	34.91	42	1.20 (0.87-1.63)
	Females	2.29	6	2.62 (0.96-5.71)	-	<6	-
	Males	126.02	228	**1.81 (1.58-2.06)**			
Manufacturing	Both	2,164.05	4,420	**2.04 (1.98-2.10)**	650.22	948	**1.46 (1.37-1.55)**
	Females	262.36	536	**2.04 (1.87-2.22)**	107.99	149	**1.38 (1.17-1.62)**
	Males	1,901.69	3,884	**2.04 (1.98-2.11)**	542.23	799	**1.47 (1.37-1.58)**
Construction	Both	925.18	2,452	**2.65 (2.55-2.76)**	239.77	482	**2.01 (1.83-2.20)**
	Females	26.97	65	**2.41 (1.86-3.07)**	9.88	15	1.52 (0.85-2.50)
	Males	898.21	2,387	**2.66 (2.55-2.77)**	229.89	467	**2.03 (1.85-2.22)**
Trade	Both	1,745.13	2,665	**1.53 (1.47-1.59)**	472.68	556	**1.18 (1.08-1.28)**
	Females	338.24	544	**1.61 (1.48-1.75)**	126.03	146	1.16 (0.98-1.36)
	Males	1,406.89	2,121	**1.51 (1.44-1.57)**	346.66	410	**1.18 (1.07-1.30)**
Transportation, Communication, Other Utilities	Both	701.07	1,187	**1.69 (1.60-1.79)**	208.86	324	**1.55 (1.39-1.73)**
	Females	72.53	130	**1.79 (1.50-2.13)**	30.00	47	**1.57 (1.15-2.08)**
	Males	628.54	1,057	**1.68 (1.58-1.79)**	178.86	277	**1.55 (1.37-1.74)**
Community, Business, and Personal Service	Both	2,111.99	4,056	**1.92 (1.86-1.98)**	637.30	881	**1.38 (1.29-1.48)**
	Females	908.67	1,605	**1.77 (1.68-1.85)**	348.92	484	**1.39 (1.27-1.52)**
	Males	1,203.32	2,451	**2.04 (1.96-2.12)**	288.38	397	**1.38 (1.24-1.52)**
Public Administration and Defence	Both	647.84	709	**1.09 (1.02-1.18)**	204.58	281	**1.37 (1.22-1.54)**
	Females	169.78	192	1.13 (0.98-1.30)	67.95	93	**1.37 (1.10-1.68)**

Industry (Division Level)	Sex	Emergency Department Visits			Hospitalizations		
		Exp	Obs	SIR[1,2] (95% CI)	Exp	Obs	SIR[1,2] (95% CI)
	Males	478.06	517	1.08 (0.99-1.18)	136.63	188	**1.38 (1.19-1.59)**

Abbreviations: CI = confidence interval; Exp = expected cases; Obs = observed cases; SIR = standardized incidence ratio

[1] Overall findings adjusted for sex, age, calendar year, and health region. Sex-specific findings adjusted for age, calendar year, and health region.

[2] Bolded values denote statistically significant SIRs above 1. Italicized bolded values denote statistically significant SIRs below 1.
