# Supplementary material for: Occupational patterns of opioid-related harms comparing a cohort of formerly injured workers to the general population in Ontario, Canada
Source: Can J Public Health. 2024 Apr 24;115(6):851–61. doi: 10.17269/s41997-024-00882-w (PMC11638427; doi:10.17269/s41997-024-00882-w)
Supplement: Supplementary file 2 — Supplementary file2 (PDF 78.5 KB) [file 41997_2024_882_MOESM2_ESM.pdf]

SUPPLEMENTAL FIGURE

Occupational Patterns of Opioid-Related Harms Comparing a Cohort of Formerly Injured Workers to the General Population in Ontario, Canada

Carnide N, Feng G, Song C, Demers PA, MacLeod JS, Sritharan J.

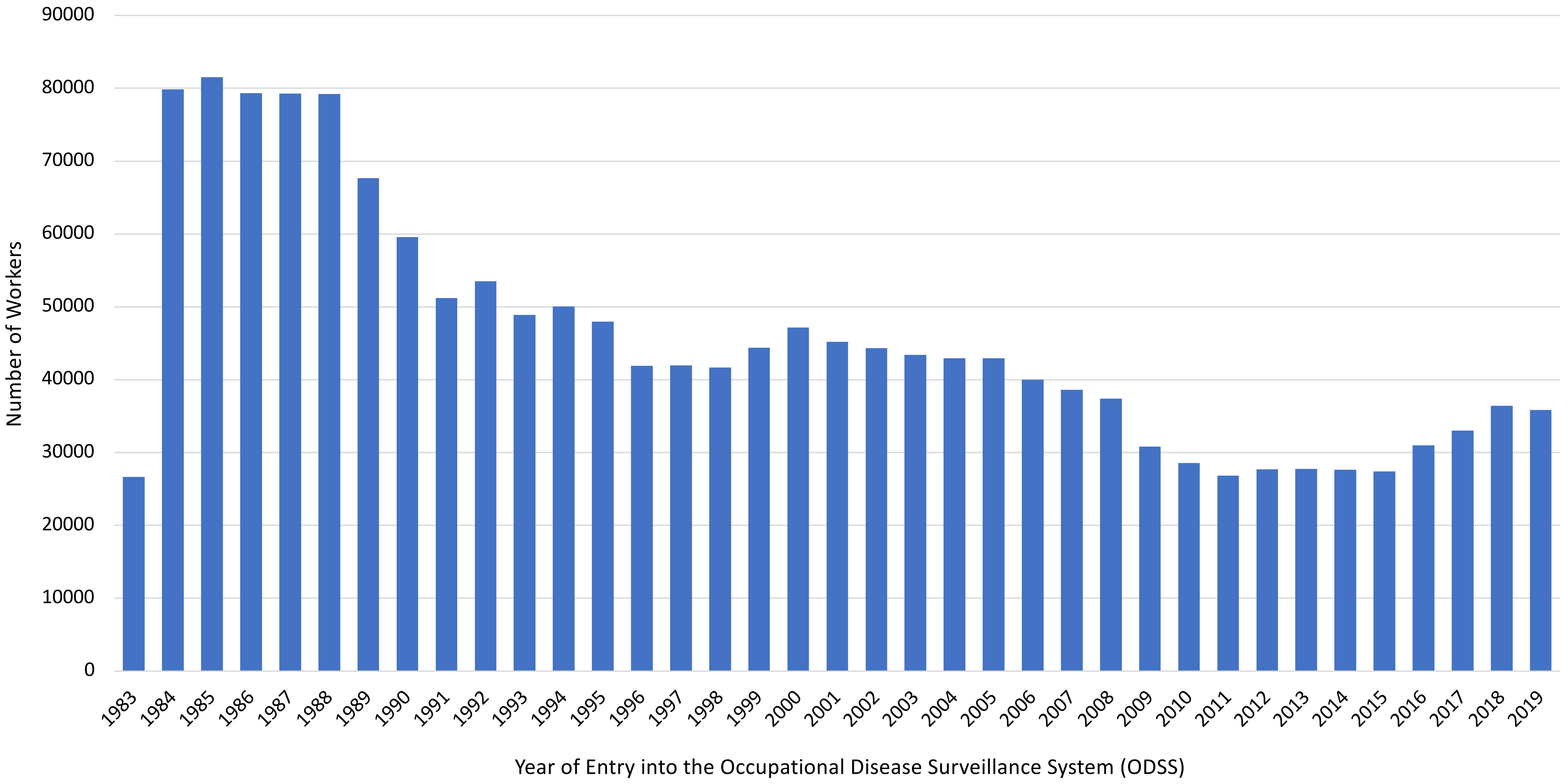

Supplemental Fig. 1 Number of workers added to the Occupational Disease Surveillance System (ODSS) by year of entry
